# Stabilizing Defect Visibility Under Overexposure in Fringe-Based Imaging via *γ* Nonlinearity Analysis

**DOI:** 10.3390/s26072032

**Published:** 2026-03-25

**Authors:** Xiaolong Ma, Xiaofei Wang, Ruizhan Zhai, Zhongqing Jia, Wei Zhang, Bing Zhao, Chen Guan

**Affiliations:** Qilu University of Technology (Shandong Academy of Sciences), Laser Institute, Shandong Academy of Scienses, China-Belarus Belt and Road Joint Laboratory on Intelligent Perception in Extreme Environments, Shandong Key Laboratory of Optoelectronic Sensing Technologies, National-Local joint Engineering Laboratory for Energy and Environment Fiber Smart Sensing Technologies, 3501 Daxue Road, Changqing District, Jinan 250353, China; 15653990653@163.com (X.M.); wxf301302@qlu.edu.cn (X.W.); zrz@vip.sdlaser.cn (R.Z.); jiazhongqing@vip.sdlaser.cn (Z.J.);

**Keywords:** fringe projection profilometry, gamma nonlinearity, computational imaging, defect saliency, overexposure analysis

## Abstract

Phase-shifting fringe projection (PSFP) is widely used in industrial inspection and three-dimensional measurement, where γ nonlinearity of the projector–camera system is traditionally treated as a phase-error source to be calibrated or compensated. In this work, γ nonlinearity is reinterpreted from an imaging perspective and shown to act as a statistical distortion mechanism that reshapes modulation stability, overexposure behavior, and defect saliency in fringe-based imaging. Building on the intrinsic DC–AC decomposition of phase-shifting demodulation, we analyze how γ nonlinearity interacts with fringe modulation and frequency-selective transfer. An analytical model reveals that γ nonlinearity simultaneously suppresses the fringe fundamental and introduces harmonic leakage, leading to systematic compression of mean modulation contrast in high-brightness regions. As a result, γ correction does not necessarily enhance mean-based defect contrast and may even reduce it, contrary to common intuition. We further demonstrate that the primary benefit of γ correction lies in statistical stabilization rather than contrast amplification. By introducing modulation-domain saliency formulations and a frequency-domain harmonic energy ratio, a physical link is established between γ nonlinearity, overexposure, and defect separability. Controlled experiments on highly reflective sheet-metal specimens confirm that while mean-contrast- and SNR-based saliency metrics often decrease after γ correction, separability-based metrics consistently improve due to reduced nonlinear- and saturation-induced variance. Cross-channel and cross-condition analyses further show that modulation and reflectance images respond differently to γ correction, yet metric-level separability exhibits consistent improvement across channels. These results clarify the true role of γ correction in fringe-based inspection and provide theoretical insight and practical guidance for robust defect imaging under nonlinear and near-overexposure conditions.

## 1. Introduction

Optical inspection of highly reflective industrial surfaces [1,2,3,4] plays a critical role in quality control across manufacturing sectors such as automotive body assembly, aerospace structures, and precision metal components. In these applications, surface defects including scratches, scuffs, dents, and micro-structural irregularities often coexist with strong specular reflections and large dynamic-range variations. As a result, captured images frequently suffer from local overexposure, nonlinear intensity compression, and unstable image statistics, which jointly degrade defect visibility and detection reliability.

In practice, these imaging degradations cannot be reliably eliminated through conventional exposure control or illumination adjustment alone. For large or complex workpieces, the coexistence of low-reflectance background regions and high-reflectance defect-adjacent areas forces the imaging system to operate close to nonlinear or near-saturation regimes [2,3]. Importantly, defect regions are often spatially correlated with intensity peaks, making them particularly sensitive to nonlinear response and demodulation instability. This coupling between surface reflectance, nonlinear imaging response, and defect visibility poses a fundamental challenge for robust optical inspection.

PSFP is one of the most mature and widely adopted structured-light techniques for industrial three-dimensional measurement and surface inspection [5,6,7,8,9,10,11,12]. By projecting sinusoidal fringe patterns and performing pixel-wise phase demodulation, PSFP enables dense surface reconstruction with high accuracy and robustness to weak texture. Beyond geometric measurement, fringe-based methods have also been employed to enhance defect visibility through modulation analysis, dark-field-like imaging, and multi-exposure or high-dynamic-range (HDR) strategies.

To address overexposure and dynamic-range limitations on shiny surfaces, a variety of HDR fringe projection techniques have been proposed, including multi-intensity projection and exposure scheduling [5,6,7], combined shape–texture acquisition and reflectance-aware strategies [8,9], as well as modulation-based enhancement methods that exploit the amplitude of the fringe fundamental [10,11]. Comprehensive reviews further summarize advances in phase-shifting algorithms and HDR-oriented fringe projection pipelines [12]. While these approaches improve robustness under challenging reflectance conditions, their effectiveness is often evaluated empirically, and the underlying imaging mechanisms governing defect visibility remain insufficiently understood. In particular, the interaction between system nonlinearity, fringe demodulation, and defect saliency has not been systematically modeled at the imaging–statistical level.

In practical projector–camera systems, the radiometric response is inherently nonlinear and is commonly modeled using a power-law gamma function. Extensive studies have demonstrated that gamma nonlinearity distorts ideal sinusoidal fringe patterns, introduces higher-order harmonic components, and degrades phase accuracy as well as three-dimensional reconstruction precision. As summarized in comprehensive reviews on PSFP algorithms [12], these nonlinear effects manifest as phase deviation, fringe waveform distortion, and systematic reconstruction errors. Consequently, most existing studies treat gamma nonlinearity primarily as an error source to be calibrated or compensated, with performance metrics largely focused on phase accuracy, fringe sinusoidality, or geometric reconstruction fidelity [13,14,15,16,17,18].

A variety of calibration and compensation strategies have been proposed to mitigate gamma-induced errors, including response curve fitting [13,14], dual-frequency or multi-pattern correction schemes [15,16], direct nonlinearity recognition from phase maps [17], and adaptive or high-precision gamma compensation methods for improved fringe pattern generation [18]. Related studies in fringe reflection and phase-shifting interferometry further confirm that nonlinear camera and projector responses constitute a dominant source of phase error in precision measurement systems [19,20]. Under these formulations, gamma nonlinearity is largely regarded as a deterministic distortion that can be compensated once the system response is accurately characterized.

However, under high-reflectance or near-overexposure conditions, gamma nonlinearity influences not only phase accuracy but also the statistical properties of the demodulated images themselves. Even in the absence of hard pixel saturation, nonlinear intensity mapping can compress modulation amplitudes, enhance harmonic leakage, and inflate local statistical fluctuations. Prior analyses of partial saturation and intensity clipping have mainly focused on phase deviation and phase retrieval accuracy [21,22,23], while their implications on modulation stability, variance amplification, and defect separability have received comparatively limited attention at the imaging–statistical level, which will be analytically addressed in Section 3. As a result, it remains unclear why gamma correction, despite being widely adopted and theoretically well-founded, often leads to limited or inconsistent visual contrast improvement in defect inspection tasks.

These observations suggest that gamma nonlinearity should be reconsidered from an imaging-level and statistical perspective, rather than being viewed solely as a phase error source. In particular, understanding how gamma-induced nonlinear distortion reshapes the distribution and stability of modulation signals is essential for explaining defect visibility behavior under nonlinear and near-overexposure imaging conditions. Existing γ calibration/compensation studies primarily evaluate performance using phase deviation, fringe sinusoidality, or reconstruction accuracy. By contrast, how γ correction reshapes the demodulated modulation/reflectance statistics that govern defect visibility and defect–background separability has not been explicitly modeled and validated.

A key but often under-emphasized property of PSFP is its intrinsic decomposition of the captured signal into physically distinct components. Through standard phase-shifting demodulation, a DC component associated with surface reflectance and illumination conditions, and an AC component corresponding to the amplitude of the fringe fundamental (commonly referred to as the modulation), are obtained simultaneously from the same acquisition [10,12]. From a signal-processing perspective, this process can be interpreted as a DC–AC separation in the spatial frequency domain, where the fundamental fringe component is isolated from the background intensity distribution.

The modulation component is not merely an auxiliary quantity for phase estimation. Early studies on modulation measurement profilometry have shown that the fringe amplitude itself carries meaningful information about surface structure, local scattering behavior, and demodulation reliability [10]. Subsequent fringe-based imaging and relief visualization methods further demonstrate that modulation-related channels respond differently to surface morphology and illumination variations than conventional reflectance images [11]. As a result, modulation images exhibit distinct sensitivity to surface microstructure and imaging instability, making them particularly relevant for defect-related analysis.

Consequently, modulation images respond differently to nonlinear intensity mapping and saturation effects than reflectance images. While reflectance primarily reflects the DC radiometric response of the imaging chain, modulation directly depends on the preservation of the fringe fundamental after projection, reflection, and capture. This observation suggests that PSFP should be interpreted not only as a geometric measurement technique, but also as a modulation–demodulation-driven computational imaging framework that naturally produces multiple image channels with complementary physical meanings [12].

Within this framework, gamma nonlinearity reshapes the spectral composition and statistical distribution of the modulation signal through fundamental attenuation and harmonic leakage, as extensively reported in gamma-related phase-error and fringe non-sinusoidality analyses [12,13,14,15,16,17,18]. Its impact is therefore spatially selective and operating-point-dependent, being significantly amplified in high-brightness or near-saturation regions while remaining limited in background areas. This mechanism provides a physically grounded explanation for the frequently observed inconsistency between visual contrast enhancement and quantitative defect detectability under gamma correction, motivating the need for an imaging-level and statistical reinterpretation of gamma nonlinearity.

Despite extensive studies on gamma nonlinearity in fringe projection, several important gaps remain. First, most analyses are phase-centric and do not distinguish between the channel-dependent responses of reflectance and modulation components. Second, defect visibility is often evaluated using heuristic contrast or signal-to-noise metrics without explicit consideration of nonlinear-induced statistical instability. Third, overexposure and gamma nonlinearity are typically treated as separate issues, even though they are intrinsically linked at high-intensity operating points.

This work addresses these gaps by reinterpreting gamma nonlinearity as a statistical distortion mechanism acting on fringe-based imaging rather than merely as a geometric error source. By analyzing how gamma nonlinearity reshapes modulation distributions, amplifies harmonic leakage, and alters defect separability, we aim to clarify the true role of gamma correction in high-reflectance surface inspection. Importantly, this study focuses on imaging-level observability and statistical stability, rather than proposing a specific defect detection algorithm.

To avoid ambiguity, this work does not address defect detection based on raw carrier fringes or time–frequency analysis, nor does it focus on shape-defect identification via three-dimensional reconstruction errors or phase discontinuities. Instead, the emphasis is placed on modulation-domain imaging behavior and its statistical implications under nonlinear and near-overexposure conditions.

The main contributions of this work are summarized as follows:

1. Statistical interpretation of gamma nonlinearity: Gamma nonlinearity is reformulated as a statistical distortion mechanism that governs modulation stability, harmonic leakage, and defect separability, rather than as a simple phase-error source.
2. Channel-aware fringe imaging analysis: By exploiting the intrinsic DC–AC decomposition of phase-shifting demodulation, the distinct responses of reflectance and modulation images to gamma nonlinearity are systematically analyzed within a unified framework.
3. Modulation-domain defect saliency modeling: An analytical model is developed to explain how gamma nonlinearity simultaneously suppresses the fringe fundamental and enhances higher-order harmonics, leading to contrast compression and variance inflation in high-brightness regions.
4. Clarification of the role of gamma correction: Through controlled experiments under fixed system parameters and varying specimen reflectance and local operating-point conditions, it is demonstrated that the primary benefit of γ correction lies in statistical stabilization and improved separability, rather than in mean contrast enhancement.
5. Unified statistical evaluation across channels: A representation-agnostic saliency formulation is introduced, enabling consistent quantitative comparison of defect visibility across modulation and reflectance images.

The remainder of this paper is organized as follows. Section 2 presents the imaging model and system description under non-ideal industrial conditions. Section 3 presents the theoretical analysis of gamma-induced modulation distortion, harmonic behavior, and defect saliency. Section 4 validates the theoretical findings through controlled experiments on highly reflective industrial specimens. Section 5 concludes the paper and discusses implications for robust fringe-based inspection under nonlinear imaging conditions.

## 2. Imaging Model and System Description (Non-Ideal Industrial System)

### 2.1. System Composition and Uncontrollable Radiometric Response

The imaging system considered in this work consists of a digital projector and an industrial camera operating in a PSFP configuration, which is widely adopted in industrial three-dimensional measurement and surface inspection systems [5,6,7,8,9,10,11,12]. Unlike idealized imaging models commonly assumed in laboratory conditions, the effective radiometric response of a practical projector–camera system is jointly determined by multiple coupled and largely uncontrollable factors, including the projector output characteristics, camera sensor response, exposure settings, quantization effects, electronic gain, and embedded digital image processing pipelines [12,19,20].

As a result, the intensity recorded by the camera does not represent a direct linear mapping of the projected fringe signal. Instead, it reflects a composite system response that integrates optical projection, surface reflection, sensor exposure, analog-to-digital conversion, and internal signal processing. Even when identical fringe patterns are projected, the captured intensity distributions may vary significantly across different surface regions and imaging conditions, especially for high-reflectance or near-specular materials [1,2,3,4].

In industrial inspection scenarios, such variations cannot be fully controlled or eliminated through exposure adjustment alone. For large or complex workpieces, low-reflectance background regions often coexist with highly reflective areas adjacent to defects, forcing the imaging system to operate close to nonlinear or near-saturation regimes [2,3]. Importantly, defect regions are frequently spatially correlated with intensity peaks, making defect visibility particularly sensitive to radiometric non-idealities.

Therefore, throughout this work, the projector–camera pair is treated as a non-ideal, condition-dependent imaging system, rather than as a perfectly calibrated linear device. This viewpoint is consistent with the imaging challenges discussed in the introduction and forms the foundation for reinterpreting γ nonlinearity as an intrinsic system response rather than a removable modeling artifact.

### 2.2. Phase-Shifting Fringe Projection as a DC–AC Imaging Process

In a typical PSFP system, the fringe intensity projected onto the object surface can be expressed as

(1) $$I_{n}\left(x,y\right)=I_{r}\left(x,y\right)+I_{m}\left(x,y\right)\cos\left(\phi \left(x,y\right)+\delta_{n}\right),$$

where $I_{r}\left(x,y\right)$ denotes the background (DC) component associated with surface reflectance and illumination conditions, $I_{m}\left(x,y\right)$ represents the fringe modulation amplitude, ϕ(x,y) is the phase related to object geometry, and $\delta_{n}$ is the imposed phase shift. Using standard multi-step phase-shifting algorithms, the reflectance and modulation components can be explicitly separated at the pixel level. In classical three-step phase-shifting methods [12,24,25], three fringe images with phase shifts $\delta_{n}=2\left(n-1\right)\pi /3$ (n=1,2,3) are captured.

The background and modulation intensities are then computed as

(2) $$I_{r}=\frac{I_{1}+I_{2}+I_{3}}{3},$$

(3) $$I_{m}=\frac{\sqrt{2}}{3}\sqrt{{\left(I_{1}-I_{2}\right)}^{2}+{\left(I_{2}-I_{3}\right)}^{2}+{\left(I_{3}-I_{1}\right)}^{2}}.$$

From a signal-processing and frequency-domain perspective, Equations (1)–(3) naturally correspond to a DC–AC decomposition. The reflectance image $I_{r}$ is dominated by the zero-frequency component and mainly describes macroscopic reflectance and illumination distribution, whereas the modulation image $I_{m}$ corresponds to the ±1st harmonic components and carries high-frequency information induced by surface microstructures, roughness, and local scattering [10,11].

This DC–AC decomposition is not merely a mathematical operation but directly arises from the physical process of fringe projection and phase demodulation. Consequently, PSFP should be interpreted not only as a geometric measurement technique, but also as a modulation–demodulation-driven computational imaging framework that naturally generates multiple image channels with distinct physical meanings within a single acquisition [12].

### 2.3. γ Nonlinearity as an Inherent System Response

In practical systems, the radiometric responses of projectors and cameras usually deviate from ideal linearity and are commonly modeled using a power-law gamma nonlinearity. Numerous studies have demonstrated that gamma nonlinearity distorts sinusoidal fringe modulation, introduces higher-order harmonic components in the frequency domain, and leads to phase estimation bias and three-dimensional reconstruction errors [13,14,15,16,17,18].

However, in industrial inspection systems, γ nonlinearity should be regarded as an inherent system response rather than a removable modeling error, as it cannot be fully eliminated through calibration alone.

First, the effective γ response is a composite outcome of the projector, camera, and internal signal processing pipelines, rather than a single device characteristic [12,19]. Second, the operating point of each pixel varies with local reflectance, illumination, and exposure conditions, resulting in spatially non-uniform nonlinear behavior [2,3]. Third, calibration procedures are typically performed under controlled conditions that do not fully represent the dynamic and heterogeneous environments encountered in real inspection tasks.

As a result, even after calibration or compensation, residual γ-induced nonlinear distortion persists, particularly in high-brightness or near-overexposure regions [21,22,23]. These regions are precisely where defect visibility is most critical.

Accordingly, this work does not assume that γ nonlinearity can be fully corrected or removed. Instead, γ nonlinearity is treated as an intrinsic, condition-dependent system response that reshapes the captured intensity distribution and the statistical properties of the demodulated images.

### 2.4. Imaging-Level Consequences of γ Nonlinearity

From an imaging perspective, γ nonlinearity affects the fringe-based measurement pipeline in two coupled ways. First, it suppresses the amplitude of the fringe fundamental, leading to a compression of the modulation signal in high-intensity regions. Second, it introduces higher-order harmonic components that leak into the demodulated channels, increasing variance and statistical instability [12,13,14,15,16,17,18].

Importantly, these effects occur even in the absence of hard pixel saturation. Near-overexposure operating points amplify nonlinear intensity mapping, causing modulation statistics to become highly sensitive to small perturbations in illumination and reflectance. As a result, defect regions—often coinciding with brightness peaks—exhibit increased statistical fluctuation and reduced separability from the background.

These imaging-level effects provide a physical explanation for the frequently observed phenomenon that γ correction does not necessarily enhance mean-based defect contrast and may even reduce it. As will be analytically shown in Section 3.3 and quantitatively validated in Section 4.5, the primary benefit of γ correction lies in statistical stabilization rather than direct contrast amplification.

### 2.5. Modeling Philosophy and Scope

Based on the above considerations, this work adopts a modeling philosophy that explicitly acknowledges system non-idealities and uncontrolled operating conditions. Rather than pursuing perfect radiometric linearization, we focus on understanding how γ nonlinearity reshapes modulation distributions, amplifies harmonic leakage, and influences defect visibility from a statistical perspective.

Accordingly, the analysis in this paper operates at the imaging level and does not aim to improve phase accuracy or three-dimensional reconstruction precision. Methods based on carrier fringe analysis or time–frequency representations [26], shape-defect detection via three-dimensional reconstruction errors [27], or phase-domain anomaly analysis [28] fall outside the scope of this study.

Instead, the emphasis is placed on modulation-domain imaging behavior under nonlinear and near-overexposure conditions, which forms the foundation for the analytical modeling in Section 3 and the experimental validation in Section 4.

## 3. Impact of γ Nonlinearity on Modulation, Overexposure, and Defect Visibility

### 3.1. Fringe-Structured Computational Imaging Perspective

As discussed in Section 1 and Section 2, phase-shifting fringe projection (PSFP) should not be regarded solely as a geometric measurement technique, but rather as a modulation–demodulation-driven computational imaging process that inherently produces multiple image channels with distinct physical meanings [10,11,12]. Through standard phase-shifting demodulation, the captured fringe intensity is decomposed into a reflectance (DC) component and a modulation (AC) component associated with the fundamental fringe frequency.

In practical industrial systems, the effective γ nonlinearity arises from the combined response of the projector, camera sensor, quantization, and embedded digital processing pipelines, and cannot be fully eliminated through calibration alone [12,19,20]. From an imaging perspective, the primary concern is therefore not whether γ nonlinearity can be perfectly corrected, but how it reshapes the modulation signal under high-intensity and near-overexposure operating points, where defect visibility becomes unstable.

Within this framework, γ nonlinearity should be interpreted not merely as a phase-error source, but as a statistical distortion mechanism acting on fringe modulation distributions through fundamental attenuation, harmonic leakage, and operating-point-dependent variance inflation. This interpretation directly corresponds to the statistical reinterpretation of γ nonlinearity outlined in Section 1 and forms the basis for the analytical modeling developed in this section.

### 3.2. Relationship Between γ Nonlinearity and Defects

#### 3.2.1. Ideal Fringe Model and Defect-Induced Modulation Perturbation

To establish a physically interpretable model, we begin with an ideal one-dimensional sinusoidal fringe signal

(4) $$I\left(x\right)=I_{0}\left[1+k\cos\left(\omega x+\phi \right)\right],$$

where $I_{0}$ is the mean intensity, 0<k<1 is the normalized fringe contrast, and ω is the spatial carrier frequency. For analytical convenience, the normalized form is written as

(5) $$\tilde{I}\left(x\right)=1+k\cos\left(\omega x\right)$$

Local defects are modeled as perturbations to the modulation depth,

(6) $$\tilde{k}\to k+\Delta k$$

where Δk denotes the defect-induced change in local modulation depth. This perturbation is physically consistent with local variations in surface microstructure, roughness, or scattering behavior [10,11]. The corresponding fringe signal in a defective region is therefore

(7) $$\tilde{I_{d}}\left(x\right)=1+\left(k+\Delta k\right)\cos\left(\omega x\right)$$

Equation (6) models the effect of a local defect as a perturbation of the modulation depth. A detailed derivation and residual analysis of the perturbation model are provided in Appendix A. The detailed derivation of this perturbation model, together with its equivalent multiplicative representation and residual analysis, is provided in Appendix A, where the additive perturbation form $\tilde{k}=k+\Delta k$ and its equivalent relative (multiplicative) representation $\tilde{k}=k\left(1+\xi \right)$ are obtained under a weak-perturbation assumption.

In practical inspection scenarios, however, the physical severity of defects (e.g., scratch depth, roughness variation, or scattering changes) is difficult to quantify using a unified parameter across heterogeneous defect categories. Therefore, instead of directly using physical perturbation quantities such as Δk, this work adopts an imaging-level severity representation based on statistical detectability.

Specifically, Section 3.3.2 introduces the background-noise-normalized saliency metric (${\tilde{V}}_{\mathrm{snr}}$), which serves as an operational proxy for defect severity in the modulation domain. The connection between the modulation perturbation model in Equation (6) and the statistical detectability metrics used in the experiments is further clarified through the analysis in Section 3.3.2 and Appendix A. This interpretation clarifies that Equation (6) does not assume a specific physical defect model but rather provides a generic perturbation framework linking modulation changes to statistically measurable defect detectability.

#### 3.2.2. Spectral Decomposition Induced by γ Nonlinearity

The radiometric response of the projector–camera system is commonly modeled using a power-law γ nonlinearity,

(8) $$\tilde{I_{\gamma }}\left(x\right)=\tilde{I}{\left(x\right)}^{\gamma }.$$

The power-law radiometric response model is widely used to describe the nonlinear intensity response of imaging systems, including cameras, display devices, and projector–camera systems [29,30,31].

The modulation depth *k* represents the normalized fringe contrast and is therefore bounded within the interval 0<k<1 in practical fringe projection systems. For moderate modulation depth satisfying |k|<1, a second-order expansion with respect to *k* gives

(9) $$\tilde{I_{\gamma }}\left(x\right)=1+\gamma k\cos\left(\omega x\right)+\frac{\gamma \left(\gamma -1\right)}{2}k^{2}\cos^{2}\omega x+R_{\gamma }.$$

where the neglected higher-order term satisfies

$$R_{\gamma }=O\left(k^{3}\right).$$

Using the trigonometric identity $\cos^{2}\left(\omega x\right)=\frac{1+\cos\left(2\omega x\right)}{2}$, Equation (9) becomes

(10) $${\tilde{I}}_{\gamma }\left(x\right)=1+\gamma k\cos\left(\omega x\right)+\frac{\gamma \left(\gamma -1\right)}{4}k^{2}+\frac{\gamma \left(\gamma -1\right)}{4}k^{2}\cos\left(2\omega x\right)+R_{\gamma }.$$

Equations (9) and (10) show that γ nonlinearity simultaneously attenuates or rescales the fundamental component and introduces a second-harmonic term. The remainder $R_{\gamma }$ quantifies the truncation error of the second-order expansion and remains negligible when |k|<1 and the imaging system operates in a non-saturated radiometric regime.

Equation (10) explicitly shows that γ nonlinearity:

(i) Scales the fundamental component by a factor proportional to γ;
(ii) Introduces a second-harmonic component whose amplitude is proportional to γ(γ−1);
(iii) Degrades fringe sinusoidality as γ deviates from unity.

These effects provide a direct mechanism for pseudo-fringe artifacts and harmonic leakage in digitally projected fringe patterns, which have been widely reported in γ-related fringe projection analyses [13,17].

It should be emphasized that Equation (10) does not imply that γ correction necessarily enhances defect saliency defined by mean modulation contrast. On the contrary, for γ>1, the above analysis predicts a systematic compression of modulation variations in high-brightness operating regions, where defects are often spatially correlated with intensity peaks (Section 1 and Section 2). Consequently, the potential benefit of γ correction lies not in mean enhancement, but in suppressing nonlinear-induced fluctuations and stabilizing the statistical structure of defect responses, which will be quantitatively evaluated in Section 4.

### 3.3. Modulation-Domain Defect Saliency Under γ Nonlinearity

Based on the spectral analysis in Section 3.2, γ nonlinearity affects defect visibility through both mean modulation compression and variance inflation. This section quantitatively analyzes the impact of γ nonlinearity on defect saliency in the modulation domain, with the objective of establishing a causal and physically grounded saliency model that links γ-induced spectral distortion to defect separability under near-overexposure conditions.

#### 3.3.1. Mean-Based Modulation-Domain Saliency Under γ Nonlinearity

In machine vision and industrial inspection, defect saliency (or defect visibility) describes the degree to which defect regions stand out relative to their local background. Early perceptual studies established that the visibility of periodic structures and texture anomalies is closely related to local contrast [32]. In industrial applications, defect detectability is often quantified using brightness contrast, texture energy, or statistical differences [33,34,35], while frequency-domain features such as Gabor responses have also been employed.

With the development of visual saliency theory, attention-based models have been introduced to highlight conspicuous regions in complex scenes [36,37,38]. However, these approaches typically operate in the intensity or gradient domain and do not explicitly consider imaging nonlinearity.

In fringe-structured computational imaging, defects do not manifest merely as simple brightness variations but instead perturb the stability of fringe modulation and demodulation [14,15]. Consequently, defect visibility must be analyzed within the modulation domain rather than in raw intensity images. To quantify this, we define a region-averaged modulation-domain defect saliency $V_{c}$ as follows:

(11) $$V_{c}=\frac{\mu_{m,d}-\mu_{m,b}}{\mu_{m,b}+\varepsilon },$$

where $\mu_{m,d}$ and $\mu_{m,b}$ denote the mean modulation amplitudes within the defect and background regions, respectively, and ε is a small constant to ensure numerical stability. Under the normalized ideal model in Equation (5), the modulation amplitude is equal to the fringe contrast *k*. Therefore, $V_{c}$ reduces to a direct representation of the relative modulation perturbation Δk/k in the linear, noise-free case.

However, in the presence of γ nonlinearity, the effective modulation amplitudes for the background and defect regions are reshaped according to the spectral decomposition derived in Section 3.2:

- Background Region: $I_{m,b}=\gamma k\left(1-bk^{2}\right)$, where $b=0.25\gamma \left(\gamma -1\right)$.
- Defect Region: $I_{m,d}\approx \gamma k\left(1-bk^{2}\right)+\gamma \left(1-3bk^{2}\right)\Delta k$.

By substituting these into Equation (11), the defect saliency under γ nonlinearity is obtained as:

(12) $$\tilde{V_{c}}\approx \frac{\Delta k}{k}\left[1-0.5\gamma \left(\gamma -1\right)k^{2}\right]=C_{\gamma ,k}\cdot V_{c}$$

where $C_{\gamma ,k}=1-0.5\gamma \left(\gamma -1\right)k^{2}$ acts as a contrast transfer correction factor. Equation (12) follows directly from the second-order spectral expansion in Equations (9) and (10), where higher-order residual terms are neglected under the |k|<1 assumption discussed in Section 3.2. This analytical result shows that, within the second-order approximation and for typical modulation-depth values in practical fringe projection systems, $C_{\gamma ,k}$ becomes smaller than unity when γ>1, leading to a systematic attenuation of mean-based defect saliency. This suppression is most pronounced in high-brightness and high-contrast regions, explaining why γ correction often fails to improve mean modulation contrast in practical inspection scenarios.

#### 3.3.2. Defect Severity and Separability-Based Saliency Metrics

While Equation (11) captures the separation between mean values, practical defect visibility is also governed by noise and nonlinear-induced fluctuations. To account for these factors, we introduce a hierarchical representation of statistical metrics:

Background-Noise-Normalized Saliency ${\tilde{V}}_{\mathrm{snr}}$:

(13) $${\tilde{V}}_{\mathrm{snr}}=\frac{\left|\Delta \mu \right|}{\sigma_{B}+\varepsilon }$$

This metric reflects the signal-to-noise ratio of defect responses and is widely used in industrial inspection scenarios [35].

Defect severity can be defined either in physical terms (e.g., scratch depth/width/area, roughness variation, or material-dependent scattering/reflectance changes) or in an imaging/statistical sense. In practice, a unified physical severity parameter is difficult to define across heterogeneous defect categories (scratches, scuffs, pits, stains/contamination) and materials. Therefore, in this work we adopt an imaging-level defect severity (detectability) notion, and use the background-noise-normalized saliency ${\tilde{V}}_{\mathrm{snr}}$ in Equation (13) as an operational proxy of severity. Larger ${\tilde{V}}_{\mathrm{snr}}$ indicates that the defect response is more distinguishable from background fluctuations under the given imaging condition.

Variance-Normalized Separability ${\tilde{V}}_{\mathrm{sep}}$: γ nonlinearity affects not only the background statistics but also the internal variability of defect regions. In particular, harmonic leakage and modulation collapse tend to increase the variance within defect regions, even when the mean contrast is preserved. To explicitly capture the influence of γ-induced variance inflation in both defect and background regions, a variance-normalized separability-based saliency measure is introduced as

(14) $${\tilde{V}}_{\mathrm{sep}}=\frac{\left|\Delta \mu \right|}{\sqrt{\sigma_{D}^{2}+\sigma_{B}^{2}}+\varepsilon }$$

Equation (14) jointly penalizes statistical dispersion in both defect and background regions and therefore characterizes the distributional separability of defect responses in the modulation domain.

Interpretation and hierarchical roles of the statistical metrics: While Equations (13) and (14) introduce the statistical metrics used in the experiments, it is useful to clarify their respective roles in the analysis. According to the spectral analysis in Section 3.2, γ nonlinearity introduces harmonic leakage and demodulation instability, which significantly increase intra-region variance even when mean modulation differences are preserved. Consequently, the primary benefit of γ correction lies in suppressing nonlinear-induced fluctuations and stabilizing defect statistics rather than enhancing mean contrast.

For this reason, the separability-based saliency defined in Equation (14) is particularly suitable for evaluating defect visibility under γ-nonlinear imaging conditions, as will be quantitatively validated in Section 4.

To clarify the roles of the statistical quantities introduced above, the metrics in this section can be interpreted as a hierarchical representation of defect responses in modulation images. The quantities Δμ, $\sigma_{B}$, $\sigma_{D}$ represent the first-order statistical descriptors of defect and background responses, describing respectively the mean contrast difference and the statistical variability of the two regions.

Based on these primary statistics, the background-noise-normalized metric $\tilde{V_{\mathrm{snr}}}$ in Equation (13) provides an imaging-level defect severity proxy, reflecting how strongly the defect response rises above background fluctuations. This quantity corresponds to a signal-to-noise-type detectability measure widely used in industrial inspection scenarios.

Finally, the separability metric ${\tilde{V}}_{\mathrm{sep}}$ defined in Equation (14) further accounts for the statistical spread of both defect and background distributions, providing a measure of distribution-level distinguishability.

From this perspective, the statistical metrics introduced in this section do not represent independent variables but rather form a structured hierarchy: basic statistical descriptors (Δμ, $\sigma_{B}$, $\sigma_{D}$) → detectability proxy (${\tilde{V}}_{\mathrm{snr}}$) → distribution separability (${\tilde{V}}_{\mathrm{sep}}$). This hierarchical interpretation helps clarify the roles of the metrics and avoids introducing redundant parameters in the analysis.

A detailed derivation and residual analysis of the perturbation model are provided in Appendix A.

### 3.4. Intrinsic Link Between γ Nonlinearity and Overexposure

As discussed in Section 2.2, overexposure in high-reflectance regions is not an independent phenomenon but rather an extreme manifestation of γ nonlinearity in the high-intensity regime. When the operating point approaches saturation, the nonlinear response of the projector–camera system leads to modulation collapse, harmonic amplification, and increased statistical instability.

γ correction mitigates these effects by restoring a more linear intensity mapping, thereby stabilizing the modulation response and reducing variance inflation in both defect and background regions. This intrinsic link between γ nonlinearity and overexposure explains why γ correction primarily improves statistical robustness rather than visual contrast.

### 3.5. Frequency-Domain Interpretation and Harmonic Energy Ratio

Let the local fringe signal (or modulation signal) within a spatial window be denoted as I(x). Its one-dimensional discrete Fourier transform is given by

$$F\left(\xi \right)=F\left\{I\left(x\right)\right\}$$

where ξ denotes the spatial frequency. Let the fundamental fringe frequency be *f*. The following frequency-domain energy terms are then defined

Fundamental Energy

(15) $$E_{f}=\sum_{u\in \Omega_{f}}{\left|F\left(\xi \right)\right|}^{2}$$

where $\Omega_{f}=\left[f-\Delta f,\;f+\Delta f\right]$ denotes a frequency band centered around the fundamental frequency *f*.

Second-harmonic Energy

(16) $$E_{2f}=\sum_{u\in \Omega_{2f}}{\left|F\left(\xi \right)\right|}^{2}$$

where the integration band $\Omega_{2f}=\left[2f-\Delta f,\;2f+\Delta f\right]$ is centered at 2f. The band width Δf is determined by the window size or spectral resolution.

Based on the above analysis, the effect of γ correction can be interpreted from a frequency-domain perspective as follows:

- Suppressing the second-harmonic components introduced by γ nonlinearity;
- Restoring the dominance of the fundamental component in the total fringe energy;
- Improving the consistency of modulation responses to true physical variations.

Accordingly, a fixed γ value is adopted in this work for global linearization, and a frequency-domain harmonic energy ratio is introduced as

(17) $$\rho_{2}=\frac{E_{2f}}{E_{f}}$$

This quantity serves as a local quality indicator that characterizes nonlinear distortion and overexposure severity, and provides a quantitative basis for subsequent fusion weight design and overexposure suppression.

The metric $\rho_{2}$ describes the relative energy contribution of harmonic components introduced by γ nonlinearity with respect to the effective fundamental component. In an ideal linear system, fringe energy should be predominantly concentrated at the fundamental frequency, yielding $\rho_{2}\approx 0$. When γ nonlinearity or local saturation becomes significant, second-harmonic components are amplified, leading to a noticeable increase in $\rho_{2}$.

Therefore, $\rho_{2}$ can be regarded as a unified frequency-domain indicator of fringe non-sinusoidality, overexposure risk, and modulation distortion. Unlike existing γ correction studies that primarily evaluate nonlinearity in terms of phase error or fringe sinusoidality degradation, the harmonic energy ratio introduced here directly reflects overexposure severity and modulation distortion from a frequency-domain perspective, thereby providing a physically grounded criterion for fusion weight design.

### 3.6. Interaction Between γ Nonlinearity and Fringe Density (Spatial Frequency)

Let the spatial frequency of the projected fringe be denoted as *f*. The ideal transmission of fringe modulation through the imaging system can be expressed as

(18) $$M\left(f\right)=M_{0}\cdot \mathrm{MTF}\left(f\right)$$

where $M_{0}$ denotes the intrinsic modulation amplitude of the projected fringe pattern before transmission through the imaging system, and MTF(f) represents the combined modulation transfer function of the projector–camera system. Because the optical system acts as a low-pass filter, MTF(f) generally decreases monotonically as the spatial frequency increases.

In the presence of γ nonlinearity, according to the spectral decomposition analysis in Section 3.2, the fundamental and second-harmonic components of the fringe signal are located at frequencies *f* and 2f, respectively. After accounting for both γ nonlinearity and the system frequency response, the modulation signal can be expressed as the superposition of these components

(19) $$M_{\gamma }\left(f\right)\approx \gamma \;M_{0}\;\mathrm{MTF}\left(f\right),\;E_{2f}\propto \gamma \left(\gamma -1\right)\;M_{0}^{2}\;\mathrm{MTF}\left(2f\right)$$

Since $\mathrm{MTF}\left(2f\right)<\mathrm{MTF}\left(f\right)$, when the fringe frequency increases (i.e., under high-density fringe projection), both the fundamental and harmonic components are attenuated by the system MTF, but the second-harmonic component is more strongly suppressed. This combined effect leads to:

- A reduction in fundamental energy;
- An increase in the relative contribution of harmonic components;
- An increase in the harmonic energy ratio $\rho_{2}$.

These results indicate that γ nonlinearity itself does not explicitly depend on fringe frequency. However, its observable impact is significantly amplified by fringe density. Under high-frequency fringe conditions, the combined effects of MTF attenuation, sampling limitations, and γ nonlinearity lead to more pronounced modulation degradation and pseudo-fringe artifacts.

Previous studies [15,17] have also reported that high-frequency fringe patterns are particularly sensitive to γ nonlinearity. Therefore, under dense fringe projection conditions, γ correction and frequency-domain harmonic suppression become especially critical; otherwise, phase accuracy, modulation stability, and defect saliency will be directly compromised.

### 3.7. Summary of Section 3

This section establishes an analytical framework describing how γ nonlinearity reshapes fringe modulation through fundamental suppression, harmonic amplification, and variance inflation. Rather than enhancing mean modulation contrast, γ correction primarily improves defect visibility by suppressing nonlinear-induced statistical fluctuations, which motivates the use of modulation-domain saliency measures and variance-normalized separability metrics and forms the basis for the experimental validation presented in Section 4.

## 4. Experimental Results and Analysis

### 4.1. Experimental System

**Figure 1:**
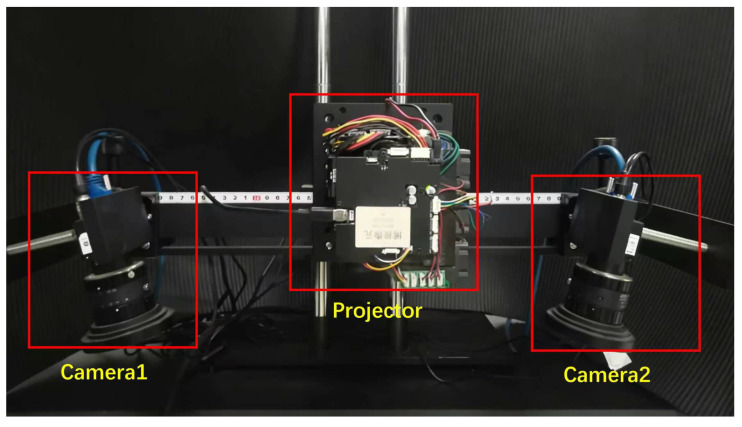
Photograph of the experimental hardware system, where the central module is labeled with “Boshi Imaging Element”, a commercial imaging component brand. The system consists of two industrial cameras (Camera1, Camera2) and a fringe projector.

The experimental setup is based on a standard phase-shifting fringe projection (PSFP) sensing configuration composed of a digital projector and an industrial camera (Figure 1). The system uses a DLP LightCrafter 4500 projector (Texas Instruments, Dallas, TX, USA, 1140 × 912 pixels) and a Hikvision MV-CS016-10UM industrial camera (Hikvision Digital Technology Co., Ltd., Hangzhou, China, 1440 × 1080 pixels, up to 249 fps). The projector and camera are synchronized via external trigger signals, enabling millisecond-level exposure control and video-rate acquisition of phase-shifted fringe patterns.

Figure 1 illustrates the system configuration used in the experiments and an expandable layout that allows optional multi-view acquisition. Such projector–camera configurations are widely used in industrial inspection and optical metrology systems. All experimental results reported in this manuscript are obtained using a single camera as specified above.

Following Equations (2) and (3) in Section 2.2, the reflectance (DC) and modulation (AC amplitude) images are demodulated from a three-step phase-shifting sequence and used throughout Section 4. In practical systems, the recorded intensity is jointly influenced by projector output response, camera sensor response, quantization, and internal processing. As a result, the effective intensity response of the projector–camera pair can be described using a power-law γ nonlinearity. This effective γ response represents the combined radiometric response of the projector, the camera sensor, and the digital processing pipeline.

Rather than attempting to eliminate this nonlinearity through calibration, this study investigates how the γ response statistically alters the distribution of fringe modulation and consequently affects defect visibility. The experimental system therefore serves as a controlled sensing platform for investigating how γ correction modifies the statistical distribution of modulation amplitudes in real fringe imaging data.

The captured fringe images are processed to obtain modulation maps, which are used for both visual inspection and quantitative statistical analysis. Representative examples of modulation images and defect regions are presented in the following sections, together with statistical metrics that quantify the changes in defect visibility under different γ correction conditions.

Unless otherwise stated, all experiments in Section 4 are conducted under the same fixed system configuration and operating point. No parameter re-selection is performed across different specimens or figures. This fixed-parameter design ensures that the observed variations in modulation stability and defect saliency originate from intrinsic γ-nonlinearity effects rather than from per-sample parameter adjustments. The purpose of the experimental section is therefore not to optimize a specific defect detection algorithm, but to analyze how γ nonlinearity reshapes the statistical properties of modulation images and consequently influences defect visibility.

### 4.2. Visualization of Modulation Changes

The influence of γ nonlinearity on fringe imaging can be intuitively understood through the visualization of modulation images. In phase-shifting fringe projection systems, the modulation image represents the amplitude of the sinusoidal fringe component and therefore reflects the local contrast of the projected fringe pattern.

Changes in the nonlinear intensity response redistribute modulation values across the image. In particular, nonlinear compression in high-intensity regions may reduce the visibility of small modulation variations, while γ correction modifies the relative contrast between defect regions and the surrounding background.

These visual effects originate from the statistical distortion mechanism analyzed in Section 3. Specifically, the nonlinear response reshapes the distribution of modulation values, affecting both the mean contrast difference and the variance structure between defect and background regions. Consequently, the statistical separability of defects in modulation images may vary depending on the effective γ response.

In fringe projection imaging, local surface defects typically influence the contrast of the projected fringes rather than introducing a simple additive intensity offset. Variations in surface microstructure, roughness, or scattering behavior modify the effective reflectance and scattering distribution, which primarily affects the amplitude of the sinusoidal fringe component. As a result, defects appear in modulation images mainly as local perturbations of fringe contrast.

This interpretation is consistent with the modulation perturbation model introduced in Equation (6). The detailed derivation of this model and its multiplicative interpretation are provided in Appendix A. In practical inspection scenarios, however, the physical severity of defects (e.g., scratch depth, roughness variation, or scattering changes) is difficult to quantify using a single physical parameter. Therefore, this work evaluates defect visibility primarily through statistical detectability metrics defined in Section 3.3, such as the background-noise-normalized saliency (${\tilde{V}}_{\mathrm{snr}}$).

Experimental observations and quantitative evaluations of these effects are presented in the following sections.

### 4.3. Pre-Experiment on the Observability of γ Nonlinearity via Fringe Density Variation

In this study, fringe density is not treated as an independent optimization parameter, nor is the purpose of this experiment to identify an optimal spatial frequency for projection. Instead, fringe density is deliberately varied as a means of probing the operating point of the imaging system, thereby exposing how γ nonlinearity manifests itself in the modulation image under different frequency-dependent transfer conditions.

From a physical perspective, changing the fringe density alters the spatial frequency of the fringe fundamental and its harmonics, which are selectively attenuated by the system modulation transfer function (MTF). While γ nonlinearity itself is independent of fringe frequency, its observable effects—such as modulation compression, harmonic leakage, and demodulation bias—are strongly conditioned by the relative transmission of the fundamental and higher-order frequency components. As a result, fringe density effectively acts as an operating-point probe, controlling the degree to which γ-induced nonlinear distortion becomes visible in the modulation domain.

Therefore, the goal of this experiment is not to compare fringe densities per se, but to establish a controlled setting in which the observability of γ nonlinearity can be systematically modulated. This pre-experiment provides essential context for the subsequent analyses in Section 4.4 and Section 4.5, where γ nonlinearity, overexposure behavior, and defect saliency are examined under conditions where nonlinear effects are either suppressed or amplified.

Fringe density (or spatial frequency) is a key system parameter. At low fringe densities (longer periods, corresponding to lower ±1st-order frequencies), the modulation signal is strong and dark-field energy is high, but fine structural details are limited. At high fringe densities, more detailed and defect-related information is encoded into higher-frequency carriers; however, due to optical transfer and sampling limitations, the modulation amplitude is significantly attenuated, resulting in a pronounced reduction in dark-field intensity. Varying fringe density thus provides an effective means to regulate dark-field strength.

To evaluate the influence of fringe density on exposure behavior in PSFP—particularly the overexposure risk on highly reflective sheet-metal surfaces—a series of controlled experiments was conducted. A large automotive sheet-metal component with high surface reflectance was used as the test object, where specular reflections easily lead to saturation. Fringe density levels were defined by fringe period: low density (period > 20 pixels), medium density (10–20 pixels), and high density (period < 10 pixels). All experiments were performed under fixed projection intensity and camera exposure time (initially set to 8000 μs) to isolate the effect of fringe density on saturation sensitivity.

The experimental procedure is as follows. First, sinusoidal fringe patterns of different densities are projected onto the sheet-metal surface and the deformed fringe images are captured. Next, the background (reflectance) and modulation images are computed using the background–modulation separation algorithm. Finally, saturation area ratios are quantified using a pixel saturation threshold (>255 for 8-bit images) to assess overexposure severity. This design ensures strict variable isolation and enables direct observation of the impact of fringe density on saturation risk.

**Figure 2:**
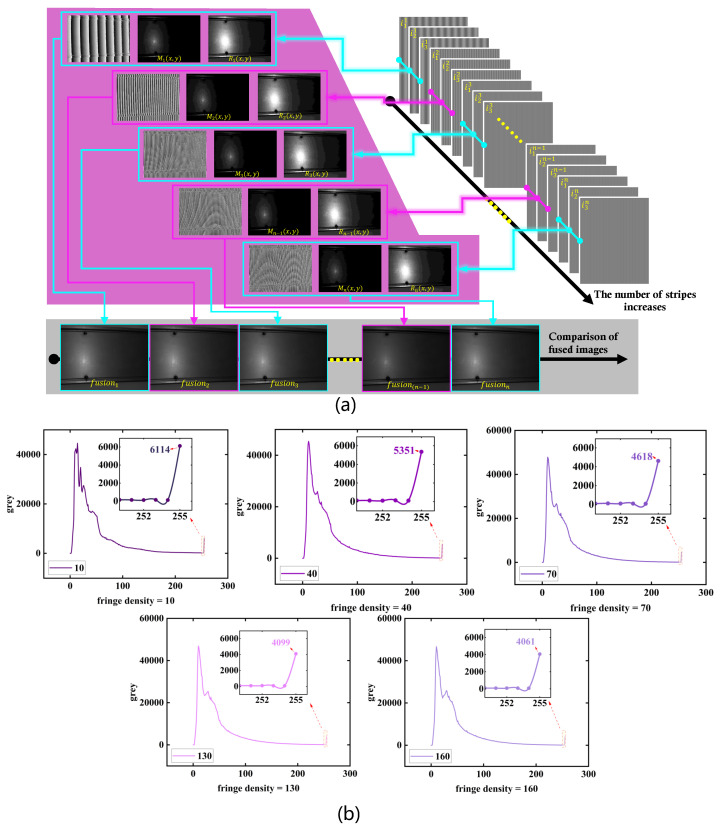
Fringe density as an operating-point probe for the observability of γ nonlinearity. (**a**) From top to bottom, the fringe density is gradually increased by varying the number of projected fringes across the projector width (1140 pixels), with the corresponding fringe numbers indicated on the right (160, 130, 100, 70, 40, and 10). For each density, the left-to-right columns show the captured fringe image, the demodulated modulation image ($I_{m}$), the reflectance image ($I_{r}$), and the originally designed projected fringe pattern. (**b**) displays the modulation histograms under different fringe densities, which intuitively reflect the effect of γ nonlinearity on the statistical distribution of modulation values.

Figure 2 shows that fringe density systematically changes the saturation tendency under fixed exposure and projection power, confirming that fringe density can be used as an operating-point probe to modulate the observability of γ-induced nonlinear distortion. Unless otherwise stated, we use a relatively high-density setting in the following experiments to avoid hard saturation under the fixed exposure, while keeping sufficient modulation energy for saliency evaluation.

Although γ nonlinearity is independent of fringe spatial frequency, changing the fringe density alters the frequency-dependent transmission of the fringe fundamental and its harmonics through the system modulation transfer function (MTF). As a result, the observable manifestations of γ nonlinearity—such as modulation compression and proximity to saturation—vary systematically with fringe density. Lower fringe densities (lower spatial frequencies) preserve stronger modulation amplitudes and are more prone to pushing high-reflectance regions toward nonlinear or near-saturation operating points, whereas higher fringe densities attenuate the modulation amplitude and suppress saturation.

Importantly, this experiment is not intended to identify an optimal fringe density, but to provide a controlled means of modulating the operating point of the imaging system, thereby exposing how γ-induced nonlinear effects become visible in the modulation domain. This operating-point probing experiment establishes the experimental basis for the analysis of γ nonlinearity, overexposure behavior, and defect saliency presented in Section 4.4 and Section 4.5.

Figure 2 shows that decreasing fringe density (i.e., increasing fringe period) significantly increases the number of saturated pixels. This phenomenon can be explained by the analysis in Section 3.6 on γ nonlinearity and fringe spatial frequency. The modulation image ($I_{m}$) corresponds to the fringe fundamental, which undergoes frequency-selective attenuation by the MTF. At low spatial frequencies, higher MTF values preserve the fundamental more effectively, leading to larger modulation amplitudes and higher peak intensities ($I=I_{r}+I_{m}$), pushing more pixels toward saturation. Conversely, high-density fringes experience stronger MTF attenuation, reducing peak intensity and saturation.

Therefore, fringe density is not treated as an optimization parameter in this work, but as a controlled means to expose and amplify γ-induced nonlinear effects for subsequent analysis. This operating-point probing experiment serves as the experimental validation of the theoretical analysis presented in Section 3.6, linking γ nonlinearity, fringe spatial frequency, and frequency-selective transfer effects to their observable impact on modulation stability. It should be emphasized that fringe density does not introduce γ nonlinearity, but only modulates its observability through frequency-selective transfer. The nonlinearity itself remains intrinsic to the projector–camera response, as modeled in Section 3. This operating-point probing experiment experimentally validates the modulation-domain analysis in Section 3.6 and directly supports the contribution in Section 1 regarding the operating-point-dependent and spatially selective nature of γ-induced modulation distortion.

### 4.4. Coupled Effects of γ Nonlinearity, Overexposure, and Defect Saliency

Building on the fringe-density analysis in Section 4.3, this section investigates imaging scenarios where surface defects coexist with high-intensity regions and strong nonlinear responses. In such cases, defect visibility cannot be explained solely by saturation ratio or mean intensity contrast. Although the contrast transfer factor C(γ,k) is analytically defined in Section 3, it is not directly measurable at the pixel level in practical imaging systems. Therefore, its experimental manifestation is evaluated through observable proxy statistics that reflect high-end modulation behavior and nonlinear spectral distortion, including percentile-based modulation measures and harmonic energy ratios. Guided by the γ-nonlinearity model in Section 3, the following analysis focuses on how these observable proxies capture the spatially selective demodulation bias and statistical instability predicted by the theory.

In such cases, defect visibility cannot be explained solely by saturation ratio. Guided by the γ-nonlinearity model in Section 3, we analyze both statistical modulation behavior and frequency-domain harmonic characteristics to validate the coupling between γ nonlinearity, fringe density, and defect saliency. Although the contrast transfer factor C(γ,k) is analytically derived, its experimental manifestation cannot be accessed directly at the pixel level. Therefore, its effect is evaluated through observable statistical and spectral proxies, including percentile-based modulation statistics (e.g., $P_{95}\left(I_{m}\right)$) and harmonic energy ratios, which are directly computable from measured images and remain robust under near-saturation conditions.

γ correction is not expected to universally enhance mean modulation contrast, focus on statistical stability and separability. In addition to separability, the background-noise-normalized saliency ${\tilde{V}}_{\mathrm{snr}}$ introduced in Section 3.3.2 is interpreted here as an imaging-level proxy for defect severity, i.e., defect detectability relative to background fluctuations. Unlike physical severity descriptors such as defect depth, width, or roughness change—which are difficult to define uniformly across heterogeneous defect categories—${\tilde{V}}_{\mathrm{snr}}$ provides an operational measure of how strongly the defect response stands out in the modulation domain under a given imaging condition. In this sense, ${\tilde{V}}_{\mathrm{snr}}$ is used to evaluate whether the defect remains sufficiently detectable after γ correction, whereas ${\tilde{V}}_{\mathrm{sep}}$ is used to assess whether the defect becomes more statistically separable from the background.

To highlight the role of γ nonlinearity under conditions where overexposure and defects coexist, a representative class of highly reflective specimens is deliberately selected in this experiment. For these specimens, defect regions are spatially co-located with intensity peaks, such that the presence of a defect itself induces local near-saturation or weak saturation.

This imaging scenario provides an ideal experimental setting for testing the theoretical predictions in Section 3 regarding γ-induced modulation degradation and harmonic enhancement. In particular, it enables direct observation of how γ nonlinearity interacts with local operating points in high-intensity regions, leading to spatially selective demodulation bias and altered defect saliency.

**Figure 3:**
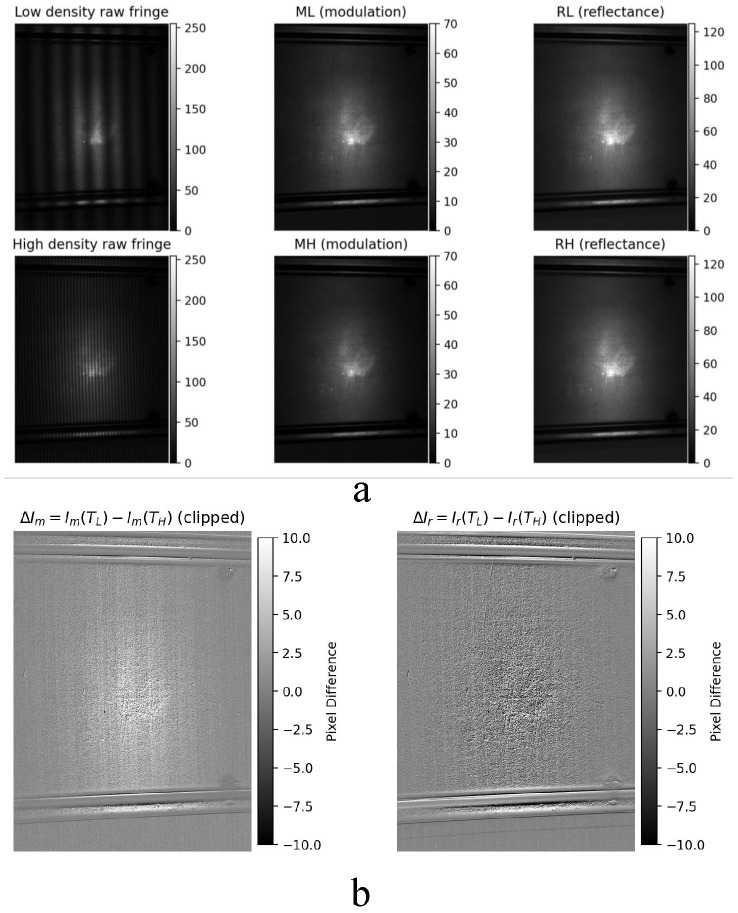
Influence of fringe density on modulation, reflectance, and nonlinear response. (**a**) Comparison under two fringe densities. The first row shows the low-density case (${\mathrm{Fringe}}_{\mathrm{number}}=20$, D20), and the second row shows the high-density case (${\mathrm{Fringe}}_{\mathrm{number}}=120$, D120). From left to right (L–R): cropped raw fringe image, modulation image $I_{m}$, and reflectance image $I_{r}$. (**b**) Difference maps of modulation and reflectance. Left: $\Delta I_{m}=I_{m}^{\mathrm{low}}-I_{m}^{\mathrm{high}}$, right: $\Delta I_{r}=I_{r}^{\mathrm{low}}-I_{r}^{\mathrm{high}}$. The difference maps are visualized using a signed gray-level representation with symmetric clipping. Notably, pronounced responses are spatially concentrated in defect regions, while background areas remain relatively stable, indicating a spatially selective demodulation bias induced by the combined effects of γ nonlinearity and fringe spatial frequency.

Two datasets with different fringe densities are employed for comparison in this experiment: a low-density fringe pattern with ${\mathrm{Fringe}}_{\mathrm{number}}=20$ (denoted as D20) and a high-density fringe pattern with ${\mathrm{Fringe}}_{\mathrm{number}}=120$ (denoted as D120). As shown in Figure 3, the first row corresponds to the low-density fringe case, while the second row corresponds to the high-density fringe case. From left to right, the cropped raw fringe image, the modulation image $I_{m}$, and the reflectance image $I_{r}$ are presented, respectively. It should be emphasized that no saturation occurs in the modulation images in either case; no hard clipping saturation occurs in $I_{m}$; near-saturation refers to the nonlinear operating regime in the raw intensity frames.

Figure 3 shows the difference maps of the modulation and reflectance images, where the left panel represents $\Delta I_{m}=I_{m}^{\mathrm{low}}-I_{m}^{\mathrm{high}}$, and the right panel represents $\Delta I_{r}=I_{r}^{\mathrm{low}}-I_{r}^{\mathrm{high}}$. These difference maps are used to highlight the response variations induced by changes in fringe density.

From the visual results, it can be observed that as the fringe density increases from D20 to D120, the overall amplitude of the modulation image decreases significantly, whereas the variation in the reflectance image is relatively limited. This trend is particularly evident in the difference maps shown in Figure 3: the modulation difference is mainly concentrated in the defect region and its surrounding neighborhood, while changes in the background region remain weak. This indicates that the combined effects of γ nonlinearity and fringe-density variation are significantly amplified in defect regions.

It is worth noting that although the overall modulation amplitude is markedly reduced under the high-density fringe condition, defect-related local structures become more clearly visible in the modulation image. This phenomenon suggests that high-density fringes do not simply suppress defect information; instead, they alter the spatial distribution of the modulation response such that local modulation disturbances induced by defects become more prominent relative to the background. Meanwhile, the defect contrast in the reflectance image also exhibits a slight increase under high-density conditions, further indicating that changes in fringe density primarily affect the global modulation amplitude, rather than the relative separability of defect regions. These observations are consistent with the analysis in Section 3, which predicts that the joint action of γ nonlinearity and spatial frequency leads to global modulation attenuation while locally enhancing relative disturbances.

From the spatial distributions in Figure 3, it can be further observed that significant variations in both the modulation difference $\Delta I_{m}$ and the reflectance difference $\Delta I_{r}$ are mainly concentrated in defect regions and along workpiece edges, while changes in non-defect, non-edge background regions remain relatively smooth. This spatial pattern indicates that the influence of γ nonlinearity and fringe-density variation on demodulation results is not globally uniform, but is instead closely related to the local imaging operating point and its spatial gradients. Defect regions and workpiece edges are typically associated with higher local reflectance, rapid geometric variations, or abrupt intensity changes, which make them more prone to approaching saturation or near-saturation regimes. Consequently, variations in the γ mapping slope and harmonic leakage effects are amplified, leading to stronger demodulation bias during fringe demodulation. In contrast, background regions are farther away from the nonlinear response threshold and are therefore less sensitive to fringe-density variation, resulting in relatively smooth modulation and reflectance changes.

For quantitative analysis, the mean modulation value, the 95th percentile $P_{95}\left(I_{m}\right)$, the saturation pixel ratio *S*, and the second-harmonic energy ratio $\rho_{2}$ (defined in Section 3.5) are introduced as statistical and frequency-domain descriptors. The results show that under the D20 fringe condition, $\mathrm{mean}(I_{m})=9.588$, $P_{95}\left(I_{m}\right)=24.935$, and the saturation ratio is approximately $S\approx 1\times 10^{-4}$. Under the D120 fringe condition, the mean modulation value decreases to 8.036 and $P_{95}\left(I_{m}\right)$ decreases to 20.955, while the saturation ratio remains at the same order of magnitude. These results indicate that the reduction in high-end modulation amplitudes is not caused by an increase in saturated pixels, but rather by fundamental-component attenuation jointly induced by γ nonlinearity and the system frequency response.

Further frequency-domain analysis shows that the second-harmonic energy ratio $\rho_{2}$ increases from $5.0912\times 10^{-2}$ under the D20 fringe condition to $5.2156\times 10^{-2}$ under the D120 fringe condition. This trend is consistent with the theoretical analysis in Section 3.5, which predicts that γ nonlinearity induces a relative enhancement of harmonic components under high spatial-frequency conditions. The result indicates that although the overall modulation amplitude decreases under high-density fringes, nonlinear distortion becomes more pronounced in the frequency domain.

Taken together, the results in Figure 3 demonstrate that in defect–high-intensity coupled scenarios, changes in defect saliency are governed primarily by the γ-induced degradation of modulation and harmonic enhancement, rather than being directly dominated by the saturation pixel ratio. The statistic $P_{95}\left(I_{m}\right)$ provides a robust characterization of the effective modulation depth within defect regions, while $\rho_{2}$ quantifies the degree of nonlinear distortion from a frequency-domain perspective. These two descriptors form a complementary set of physical diagnostic indicators, providing experimental support for subsequent γ correction and fusion-weight design.

Finally, the experimental results in Figure 3 verify that γ nonlinearity and fringe density do not exert a spatially uniform influence on demodulation outcomes. Instead, pixel-level variations in nonlinear operating points introduce a spatially selective demodulation bias, which is significantly amplified in defect regions while remaining limited in background areas. This bias directly reshapes the modulation distribution and, consequently, defect saliency.

### 4.5. Quantitative Analysis of Defect Saliency Under γ Correction

Although the absolute values of individual saliency metrics vary across specimens and defect types, the directional trend remains consistent: γ correction systematically suppresses variance-dominated instability in defect regions ($\sigma_{\mathrm{def}}↓$), while maintaining or slightly improving separability-based saliency (${\tilde{V}}_{sep}$), even when mean contrast (Δμ) decreases. This directional consistency, rather than absolute metric magnitude, is the primary experimental evidence supporting the theoretical prediction in Section 3.

According to the analytical predictions in Section 3.3, γ correction is not expected to universally enhance mean modulation contrast. Therefore, the following experiments focus on evaluating statistical stability and separability rather than visual contrast improvement. It is important to emphasize that, as predicted by the analysis in Section 3, the effectiveness of γ correction should not be evaluated solely based on mean-contrast-based saliency metrics. In particular, γ correction does not necessarily lead to an increase in mean modulation contrast, and a decrease in ${\tilde{V}}_{c}$ is not indicative of performance degradation.

Instead, the primary role of γ correction lies in suppressing nonlinear-induced fluctuations and improving the statistical stability of defect responses, as reflected by the consistent increase in the separability-based saliency ${\tilde{V}}_{sep}$. This behavior directly confirms the theoretical prediction in Section 3 that γ correction improves defect visibility predominantly through variance suppression rather than mean contrast enhancement.

**Figure 4:**
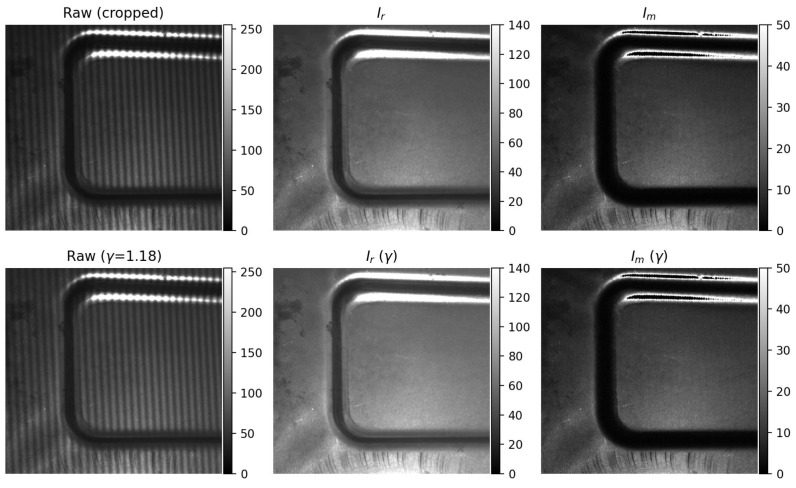
Visual relationship between γ nonlinearity and defect saliency in fringe-based imaging. The first row shows the results without γ correction (γ=1.0), and the second row shows the corresponding results after γ correction with γ=1.18. From left to right, each row presents the captured fringe image, the demodulated reflectance image $I_{r}$, and the modulation image $I_{m}$.

To verify the impact of γ nonlinearity on defect saliency, a quantitative analysis is conducted on highly reflective sheet-metal specimens in this section. A three-step PSFP method is employed, and the system γ is calibrated to approximately γ≈1.18. Since the response characteristics of the projector and the camera are not adjustable, validation is performed only at this calibrated γ value. The reflectance image and the modulation image are computed accordingly, as shown in Figure 4.

Figure 4 illustrates the relationship between γ nonlinearity and defect saliency. Overall, the visual differences are relatively subtle, which can be attributed to the moderate γ value (γ=1.18) and the correspondingly limited degree of nonlinearity. After γ correction, the modulation field $I_{m}$ becomes slightly darker, while its spatial brightness distribution appears more uniform across the field. In contrast, the reflectance field $I_{r}$ exhibits a more noticeable increase in brightness. Defect details in both $I_{m}$ and $I_{r}$ are moderately enhanced. From the perspective of optical response and frequency-domain modulation, γ correction can be interpreted as a joint redistribution of DC and AC energy. When γ>1, intensity fluctuations in high-brightness regions are nonlinearly compressed, leading to a reduction in fringe peak-to-valley contrast and modulation depth. This mechanism also explains why the bright regions near the bottom of the γ-corrected $I_{r}$ image appear more pronounced, as discussed in Section 3.3.

This figure illustrates that γ correction does not necessarily lead to a strong visual enhancement of defect contrast, especially in the modulation domain. Instead, its primary effect lies in redistributing intensity and stabilizing the modulation response, which motivates the quantitative saliency analysis presented in Section 4.5.

To further enable a quantitative analysis of γ-nonlinearity effects, traditional image-processing techniques are employed, focusing specifically on line-scratch defects, which are commonly observed on metallic surfaces. These methods do not rely on any training data; instead, they directly exploit local structural anomalies in the imaging response to extract defects. After obtaining defect masks, the saliency metrics defined in Section 3.3 are computed to quantitatively evaluate defect visibility in the imaging domain. It should be emphasized that the above segmentation and saliency computation are not intended as final detection algorithms, but rather as analytical tools for assessing separability in the imaging domain. The purpose is to verify that, under appropriate modulation-channel selection and nonlinear correction conditions, line defects already exhibit clear, stable, and quantifiable separation at the pixel level, thereby providing a favorable imaging basis for subsequent unsupervised anomaly detection or learning-based models.

**Figure 5:**
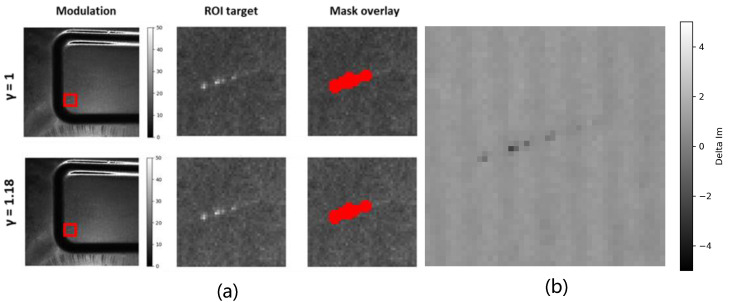
Quantitative analysis of scratch-defect saliency under γ correction. The red box marks the tiny defect on the sheet metal workpiece. (**a**) Original modulation image used for saliency evaluation. A fixed defect mask is applied to compute the modulation-domain metrics under two γ conditions (γ=1 and γ=1.18), including the mean-contrast metric $V_{c}$, the background-noise-normalized saliency ${\tilde{V}}_{\mathrm{snr}}$, and the separability metric ${\tilde{V}}_{\mathrm{sep}}$. Here, ${\tilde{V}}_{\mathrm{snr}}$ is interpreted as an imaging-level proxy for defect severity (detectability). (**b**) Difference map $\Delta I_{m}=I_{m}\left(\gamma =1.18\right)-I_{m}\left(\gamma =1\right)$, visualizing the modulation change introduced by γ correction. Although the intensity variation is small (approximately −3 to +3 gray levels), the defect region remains clearly visible in the difference image. This difference visualization highlights the subtle but structured modulation redistribution introduced by γ correction and helps reveal the effect that may appear visually weak in the original images.

Figure 5 presents the quantitative analysis of scratch-defect saliency under γ correction using a fixed defect mask. Three modulation-domain metrics are compared: the mean-contrast metric $V_{c}$, the background-noise-normalized metric ${\tilde{V}}_{\mathrm{snr}}$, and the separability-based metric ${\tilde{V}}_{\mathrm{sep}}$.

Among these, ${\tilde{V}}_{\mathrm{snr}}$ is particularly important because it serves as an imaging-level proxy for defect severity. The experimental results show that ${\tilde{V}}_{\mathrm{snr}}$ decreases slightly after γ correction (from 3.786 to 3.615), indicating that γ correction does not increase defect severity in the sense of mean-response-to-background-noise ratio. This behavior is consistent with the analytical prediction in Section 3.3: when γ>1, the mean modulation contrast is compressed by the γ-dependent transfer factor C(γ,k), so the defect response amplitude may decrease even when the image becomes statistically more stable.

By contrast, ${\tilde{V}}_{\mathrm{sep}}$ remains stable and shows a slight increase (from 0.986 to 1.001), demonstrating that the principal benefit of γ correction lies not in amplifying defect severity, but in improving defect–background separability through variance suppression. Therefore, the joint behavior of ${\tilde{V}}_{\mathrm{snr}}↓$ and ${\tilde{V}}_{\mathrm{sep}}↑$ should be interpreted as evidence that γ correction stabilizes the modulation statistics while preserving defect detectability.

The quantitative trends in Table 1 help clarify the role of ${\tilde{V}}_{\mathrm{snr}}$ as a severity proxy. After γ correction, the background fluctuation $\sigma_{B}$ decreases only slightly, whereas the defect-region fluctuation $\sigma_{D}$ decreases more noticeably, indicating that γ correction suppresses nonlinear demodulation instability inside the defect region. At the same time, the mean defect–background contrast Δμ decreases by a larger relative amount. Because ${\tilde{V}}_{\mathrm{snr}}$ is defined as a background-noise-normalized mean-response metric, it follows the reduction in Δμ and therefore decreases slightly.

This result is physically meaningful: ${\tilde{V}}_{\mathrm{snr}}$ does not measure the full distributional separability of the defect response, but rather the imaging-level severity of the defect in terms of mean-response detectability against background fluctuations. Hence, a decrease in ${\tilde{V}}_{\mathrm{snr}}$ does not imply a degradation of defect inspection performance. Instead, it indicates that γ correction redistributes modulation contrast while simultaneously suppressing nonlinear fluctuations. The corresponding increase in ${\tilde{V}}_{\mathrm{sep}}$ confirms that the net effect is improved statistical distinguishability.

The statistical results in Table 1 show that after γ correction, the modulation std in the background region $\sigma_{B}$ decreases slightly (approximately −1.8%), indicating that γ correction does reduce background fluctuations to some extent. Meanwhile, the variance within the defect region $\sigma_{D}$ decreases more noticeably (approximately −8.0%), demonstrating that demodulation instability induced by γ nonlinearity and local saturation is effectively suppressed. In contrast to these variance reductions, the mean difference between defect and background regions, $\Delta \mu =\mu_{D}-\mu_{B}$, exhibits a larger relative decrease (approximately −6.2%). This asymmetric variation is the key to understanding the different behaviors of the three saliency metrics observed in Figure 5. These results directly validate the contribution stated in Section 1 that γ correction improves defect visibility primarily through statistical stabilization and improved separability, rather than through mean contrast enhancement.

First, from the perspective of the mean-contrast-based saliency metric ${\tilde{V}}_{c}$, the experimental results clearly indicate that γ correction does not guarantee an improvement in defect saliency. On the contrary, at γ=1.18, ${\tilde{V}}_{c}$ is significantly lower than that obtained at γ=1. This trend is fully consistent with the theoretical analysis based on the analytical model presented in Section 3.3. According to the γ-dependent contrast transfer correction factor $C\left(\gamma ,k\right)$ defined in Equation (17), when γ>1, $C\left(\gamma ,k\right)<1$, and the mean modulation contrast is systematically compressed, leading to an inevitable decrease in ${\tilde{V}}_{c}=C\left(\gamma ,k\right)V_{c}$. The experimentally observed reduction in $\tilde{V_{c}}$ therefore represents a direct manifestation of this theoretical prediction under real imaging conditions, rather than an incidental numerical fluctuation.

Second, the background-noise-normalized saliency metric ${\tilde{V}}_{snr}$ likewise does not exhibit improvement after γ correction. Although γ correction slightly reduces the background noise level $\sigma_{B}$, the reduction in the defect–background mean difference Δμ is more pronounced, ultimately resulting in a net decrease in ${\tilde{V}}_{snr}$. This result indicates that γ correction is not intended to improve average-contrast SNR, and its effect should not be simply equated with conventional denoising or contrast-stretching operations. Instead, γ correction primarily alters the statistical structure of the modulation signal by reshaping its nonlinear response.

These results should be interpreted as a validation of statistical stabilization rather than visual contrast enhancement. Unlike conventional defect enhancement methods based on contrast stretching or saturation suppression [21,22,23], the observed improvement here originates from statistical stabilization rather than pixel-wise amplification.

In contrast to the two mean-dominated metrics discussed above, the separability-based saliency metric ${\tilde{V}}_{sep}$ remains stable and exhibits a slight increase after γ correction. This behavior is not driven by mean enhancement or noise suppression alone, but rather originates from changes in the internal statistical structure of the defect region. γ correction significantly compresses strong modulation fluctuations within defect regions caused by nonlinear response and local saturation, leading to a more concentrated distribution of defect responses and a substantial reduction in $\sigma_{D}$. Even with a slight decrease in Δμ, the pronounced reduction in the combined variance term in the denominator remains beneficial for improving overall defect–background separability. This effect is precisely what ${\tilde{V}}_{sep}$ is designed to capture.

Taken together, the results in Figure 5 and Table 1 lead to the conclusion that the core role of γ correction does not lie in enhancing average modulation contrast or background-normalized SNR. Instead, it lies in suppressing demodulation instability induced by γ nonlinearity and overexposure, thereby making defect responses more structured and statistically separable. This experimental observation quantitatively validates the theoretical analysis in Section 3.3 regarding the γ-dependent contrast transfer factor and the mechanism of defect saliency degradation. Therefore, γ correction should not be regarded as a conventional defect contrast enhancement technique, but rather as an imaging modulation mechanism that improves defect separability by enhancing the statistical stability of the modulation signal.

**Figure 6:**
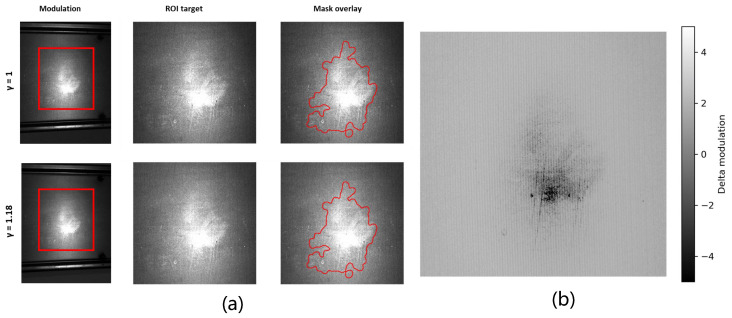
Quantitative analysis of scuff-defect saliency under γ correction under the high-density fringe condition (${\mathrm{Fringe}}_{\mathrm{number}}=120$, denoted as D120). The red box marks the defect area on the workpiece. (**a**) Modulation map $I_{m}$ obtained without γ correction (γ=1). The red rectangle indicates the scuff defect region used for quantitative evaluation. (**b**) Difference map $\Delta I_{m}=I_{m}\left(\gamma =1.18\right)-I_{m}\left(\gamma =1\right)$, visualizing the modulation change introduced by γ correction. Although the intensity variation is small (approximately −5 to +5 gray levels), the scuff defect remains clearly visible in the difference image, demonstrating that γ correction produces a measurable redistribution of modulation responses in the defect region. This difference visualization helps reveal the subtle effect of γ correction that may appear visually weak in the original images.

Furthermore, a quantitative analysis of scuff defects on the specimen shown in Figure 3 is conducted in Figure 6 to evaluate the applicability of the saliency metrics under γ correction in near-overexposure scenarios. Unlike the line-scratch defects analyzed in Figure 5, the scuff defect exhibits an area-like structure and is spatially located closer to high-intensity regions, making it more susceptible to the combined effects of γ nonlinearity and demodulation bias. As such, it provides a more challenging test case for evaluating the robustness of the saliency metrics.

The quantitative results summarized in Table 2 show that as γ increases from 1.00 to 1.18, the defect–background mean difference $\Delta \mu =\mu_{D}-\mu_{B}$ decreases slightly (from 12.206 to 11.608). Meanwhile, the statistical fluctuations in the background and defect regions remain at comparable levels: $\sigma_{D}$ decreases from 7.885 to 6.998, while $\sigma_{B}$ changes only marginally. Under these conditions, the mean-contrast-based saliency metric ${\tilde{V}}_{c}$ decreases from 1.144 to 0.918, and the background-noise-normalized SNR metric ${\tilde{V}}_{snr}$ decreases from 3.180 to 2.958. These results are consistent with the contrast compression effect in high-intensity regions predicted by the condition $C\left(\gamma ,k\right)<1$ in Section 3.3, confirming that under near-overexposure conditions, γ correction does not guarantee an improvement in mean-based saliency metrics.

It should be emphasized that no pixel-level saturation is present in the original modulation maps. The apparent saturation-like contrast in the displayed images arises solely from visualization operations—including percentile clipping (pclip), contrast stretching, and CLAHE (Contrast Limited Adaptive Histogram Equalization)—applied to facilitate reliable mask extraction and visual inspection. All quantitative metrics (${\tilde{V}}_{c},{\tilde{V}}_{snr},{\tilde{V}}_{sep}$) are computed on the original floating-point modulation maps without any clipping or enhancement. For the scuff defect, the decrease in ${\tilde{V}}_{\mathrm{snr}}$ likewise indicates that γ correction does not increase defect severity in a mean-response sense. Nevertheless, the slight increase in ${\tilde{V}}_{\mathrm{sep}}$ shows that the defect becomes more statistically separable after γ correction. This further supports the distinction between defect severity as imaging-level detectability (${\tilde{V}}_{\mathrm{snr}}$) and defect separability as distribution-level distinguishability (${\tilde{V}}_{\mathrm{sep}}$).

As a result of these visualization operations, the perceptual effect of γ correction appears relatively subtle. Nevertheless, after γ correction, a slight increase in the overall modulation intensity within the scuff region can be observed, accompanied by improved clarity of fine white scratch structures in the lower-left and lower-right areas, indicating a localized redistribution of modulation contrast rather than a global intensity amplification.

In contrast, the separability-based saliency metric ${\tilde{V}}_{sep}$ increases from 1.392 to 1.447 after γ correction, exhibiting a stable and consistent upward trend. This observation indicates that the primary role of γ correction is not to simply amplify the mean difference between defect and background regions, but rather to reshape the statistical distribution structures of defect and background responses. By doing so, γ correction effectively suppresses demodulation instability induced by nonlinear response and near-saturation effects, thereby improving defect separability in a statistical sense.

Taken together, the experimental results for line-scratch defects in Figure 5 and area-type scuff defects in Figure 6 lead to a consistent conclusion: under imaging conditions dominated by γ nonlinearity, mean-dominated saliency metrics (i.e., ${\tilde{V}}_{c}$ and ${\tilde{V}}_{snr}$) exhibit clear limitations in high-intensity or near-overexposure regions, whereas the separability-based metric ${\tilde{V}}_{sep}$ provides a more stable and reliable characterization of the defect separability improvement induced by γ correction. This result further validates the theoretical analysis presented in Section 3.3 and demonstrates that ${\tilde{V}}_{sep}$ is more suitable as a core quantitative descriptor of defect saliency under γ-nonlinearity-dominated imaging conditions.

A natural question arises: in some experiments, γ correction does not lead to a significant improvement in mean-contrast-based defect saliency metrics (such as ${\tilde{V}}_{c}$ or ${\tilde{V}}_{snr}$). What, then, is the significance of γ correction? This observation does not negate the value of γ correction; rather, it reveals the inherent limitations of mean-based saliency measures under nonlinear imaging conditions. As analyzed in Section 3.3, when γ>1, the contrast transfer correction factor C(γ,k)<1 (1) systematically compresses modulation contrast in high-intensity or near-overexposure regions. In such cases, even if the mean difference between defect and background regions Δμ increases, the accompanying statistical fluctuations ($\sigma_{B}$ and $\sigma_{D}$) are simultaneously amplified, thereby offsetting the potential gains brought by mean contrast.

In this context, the role of γ correction is not to amplify contrast, but to reshape the statistical structure of the modulation field. Although the separability-based metric ${\tilde{V}}_{sep}$ still takes the mean difference as its core component, it explicitly incorporates penalty terms for statistical fluctuations (2), enabling it to directly capture the instability introduced by γ nonlinearity and demodulation bias. For this reason, even when ${\tilde{V}}_{c}$ decreases after γ correction, ${\tilde{V}}_{sep}$ may still increase. This behavior does not indicate a failure of contrast enhancement, but rather reflects an improvement in defect separability in a statistical sense.

The consistent trends observed in Figure 5 and Figure 6, across different defect morphologies (line scratches and area-type scuff defects) and under different exposure conditions, further demonstrate that the core value of γ correction lies in suppressing statistical fluctuations induced by nonlinear response and demodulation instability, rather than simply increasing brightness or contrast. These results clarify the true functional role of γ correction in defect detection tasks.

Taken together, the observed reduction in modulation variance, the increase in harmonic energy ratio ($\rho_{2}$ defined in Section 3.5), and the divergent behavior of mean-based and separability-based saliency metrics collectively confirm the analytical predictions in Section 3.3 and Section 3.5. Taken together, the use of observable proxy statistics, fixed system parameters without per-sample tuning, and the consistent directional behavior of variance- and separability-based metrics together establish that the reported effects are intrinsic consequences of γ nonlinearity, rather than artifacts of parameter adjustment or visualization.

### 4.6. Cross-Channel Comparison Between Modulation and Reflectance Images

To further clarify the channel-dependent behavior of γ correction, a cross-channel comparison is conducted between the modulation image $I_{m}$ and the reflectance image $I_{r}$. Although both images are derived from the same physical scene, they represent fundamentally different signal components: $I_{r}$ is dominated by DC reflectance, whereas $I_{m}$ encodes the amplitude of the fringe fundamental and is therefore more sensitive to nonlinear compression and harmonic leakage.

Table 1 and Table 3 summarize the quantitative defect saliency metrics under γ correction for $I_{m}$ and $I_{r}$, respectively. Several important observations can be drawn.

First, the mean separation term Δμ exhibits opposite trends in the two channels. In $I_{r}$, Δμ slightly increases after γ correction, reflecting the redistribution of intensity levels caused by nonlinear remapping. In contrast, Δμ in $I_{m}$ decreases, indicating that γ correction suppresses saturated modulation peaks and reduces the apparent amplitude of defect responses. This opposite behavior highlights that γ correction should not be interpreted as a universal contrast enhancement mechanism.

Second, the variance structure responds differently across channels. In $I_{r}$, both background and defect variances remain largely unchanged, consistent with the DC-dominated nature of reflectance images. In $I_{m}$, however, the defect-region variance $\sigma_{D}$ shows a noticeable reduction, while the background variance changes only marginally. This confirms that γ correction primarily acts on modulation-related nonlinearities, stabilizing the demodulation process by reducing saturation-induced and harmonic-related fluctuations.

Despite these channel-dependent differences in mean and variance behavior, a consistent trend emerges at the metric level. As shown by the separability metric ${\tilde{V}}_{sep}$, both $I_{m}$ and $I_{r}$ exhibit a modest but consistent improvement after γ correction. Importantly, this improvement arises from different underlying mechanisms in the two channels: variance stabilization in $I_{m}$ and mild redistribution effects in $I_{r}$.

These results demonstrate that γ correction does not enforce statistical equivalence between modulation and reflectance images. Instead, it improves metric-level consistency across channels, even though their physical meanings and statistical responses to γ correction differ substantially. This cross-channel consistency at the saliency-metric level supports the robustness of the proposed evaluation framework under practical imaging conditions involving multiple signal channels.

While γ correction affects modulation and reflectance images through different physical mechanisms, its stabilizing effect on statistical separability is consistently observed across channels. This cross-channel comparison directly follows the DC–AC decomposition interpretation in Section 2.1 and complements the statistical reinterpretation in Section 3. This cross-channel consistency at the separability-metric level supports the channel-aware analysis and representation-agnostic evaluation strategy outlined in Section 1.

## 5. Conclusions and Outlook

This work reinterprets γ nonlinearity in PSFP not merely as a phase-error source, but as a statistical distortion mechanism that reshapes modulation stability, overexposure behavior, and defect saliency in specular-surface inspection. Building on the DC–AC demodulation framework, we analyze how γ nonlinearity interacts with fringe demodulation and frequency-selective transfer, and validate the theory using controlled experiments on highly reflective sheet-metal specimens.

Key conclusions are summarized as follows.

- γ correction is not a universal contrast enhancement mechanism.
  Section 3 predicts, through the contrast transfer factor C(γ,k), that when γ>1, the mean modulation contrast in high-brightness regions can be systematically compressed. The experiments in Section 4 confirm that mean-based saliency metrics (e.g., ${\tilde{V}}_{c}$ and ${\tilde{V}}_{snr}$) may decrease after γ correction even when defect visibility subjectively improves in localized areas.
- The primary benefit of γ correction lies in statistical stabilization rather than mean separation.
  Using fixed defect/background masks, we show that γ correction consistently reduces defect-region variance in modulation images, mitigating nonlinear- and near-saturation-induced demodulation instability. Consequently, separability-based metrics that explicitly penalize dispersion (e.g., ${\tilde{V}}_{sep}$) provide a more faithful characterization of defect detectability under nonlinear imaging conditions.
- Overexposure should be interpreted as an extreme manifestation of γ nonlinearity at the high-intensity end.
  Even without hard saturation in the modulation maps, nonlinear compression and harmonic leakage inflate local fluctuations and distort the modulation distribution. The harmonic energy ratio $\rho_{2}$ offers a complementary frequency-domain diagnostic to quantify nonlinear distortion and to explain why saliency behaviors diverge across metrics.
- γ nonlinearity is inherently channel- and condition-dependent, but metric-level improvements can be consistent across channels.
  This observation directly confirms the channel-aware imaging analysis proposed in Section 1. Cross-channel analysis shows that modulation ($I_{m}$) and reflectance ($I_{r}$) respond differently to γ correction due to their distinct physical meanings (AC amplitude vs. DC component). Nevertheless, separability-based saliency exhibits a modest yet consistent improvement across both channels, supporting the proposed channel-agnostic statistical evaluation framework.

Outlook.

Future work will extend this framework in three directions. (i) Adaptive or local γ handling: replacing a single global γ with operating-point-aware linearization to better accommodate spatially varying reflectance and near-saturation regimes. (ii) Closed-loop projection and exposure control: using modulation- and harmonic-based diagnostics (e.g., $\rho_{2}$) to guide real-time operating-point regulation for robust inspection under changing conditions. (iii) Integration with learning-based or unsupervised inspection: leveraging physically interpretable and statistically stabilized representations ($I_{m}$, $I_{r}$, and derived residuals) as more reliable inputs for anomaly detection and cross-condition generalization.

In summary, by shifting the interpretation of γ correction from “contrast boosting” to “statistical stabilization”, this work provides both theoretical insight and practical guidance for fringe-based computational imaging and robust defect inspection on specular industrial surfaces. 

## Figures and Tables

**Table 1:** Quantitative defect saliency metrics under γ correction in the modulation image $I_{m}$. The table reports the mean separation Δμ, background and defect variances ($\sigma_{B},\sigma_{D}$), and three representative saliency metrics. Among them, ${\tilde{V}}_{\mathrm{snr}}$ is treated as an imaging-level proxy for defect severity, whereas ${\tilde{V}}_{\mathrm{sep}}$ characterizes distribution-level defect separability. The results show that γ correction does not increase mean-response severity, but it improves variance structure and therefore enhances statistical separability.

γ Value	Δμ	$\sigma_{B}$	$\sigma_{D}$	$\tilde{V_{c}}$	$\tilde{V_{\mathrm{snr}}}$	$\tilde{V_{\mathrm{sep}}}$
γ=1.00	6.860	1.812	6.718	0.461	3.786	0.986
γ=1.18	6.435	1.780	6.179	0.410	3.615	1.001

Note: This table reports the mean separation $\Delta \mu =\mu_{D}-\mu_{B}$, background and defect variances ($\sigma_{B},\sigma_{D}$), and three representative saliency metrics. Although γ correction does not increase mean contrast and may even reduce Δμ, it significantly stabilizes the variance structure in modulation images, leading to a consistent improvement in the statistical separability metric $\tilde{V_{sep}}$, demonstrating that the primary role of γ correction lies in reducing condition-induced statistical instability rather than enhancing pixel-wise contrast.

**Table 2:** Quantitative saliency metrics of a representative scuff defect under γ correction (specimens shown in Figure 3 and Figure 6).

γ Value	Δμ	$\sigma_{B}$	$\sigma_{D}$	$\tilde{V_{c}}$	$\tilde{V_{\mathrm{snr}}}$	$\tilde{V_{\mathrm{sep}}}$
γ=1.00	12.206	3.839	7.885	1.144	3.180	1.392
γ=1.18	11.608	3.925	6.998	0.918	2.958	1.447

Note: All metrics are computed from the original modulation images $I_{m}$ using fixed defect and background masks. The masks are extracted from visually enhanced images solely for display and localization purposes, ensuring that the defect geometry remains unchanged during evaluation. Under this controlled setting, γ correction leads to a reduction in defect-region variance and a consistent increase in the separability metric ${\tilde{V}}_{sep}$, despite a decrease in mean contrast. This result illustrates that the benefit of γ correction arises from statistical stabilization rather than from pixel-wise contrast enhancement.

**Table 3:** Quantitative defect saliency metrics under γ correction in reflectance image $I_{r}$.

γ Value	Δμ	$\sigma_{B}$	$\sigma_{D}$	$\tilde{V_{c}}$	$\tilde{V_{\mathrm{snr}}}$	$\tilde{V_{\mathrm{sep}}}$
γ=1.00	16.139	4.201	14.610	0.265	3.842	1.062
γ=1.18	16.425	4.402	14.522	0.217	3.732	1.082

Note: In contrast to modulation images, reflectance images are dominated by DC components and exhibit only minor changes in both mean separation and variance after γ correction. Nevertheless, a slight but consistent increase in the separability metric ${\tilde{V}}_{sep}$, is observed. This result indicates that while γ correction primarily acts on modulation-related nonlinearities, its stabilizing effect on metric-level separability is not limited to a single imaging channel, supporting cross-channel consistency of the proposed saliency evaluation framework.

## Data Availability

The raw data supporting the conclusions of this article will be made available by the authors upon request.

## Appendix A. Perturbation Analysis of Modulation Depth and Imaging-Level Defect Severity

This appendix provides supplementary derivations supporting the modulation-depth perturbation model introduced in Equation (6) and clarifies the physical assumptions underlying the additive and multiplicative formulations. The appendix also explains the relationship between modulation perturbations and the imaging-level defect severity proxy used in Section 3.3.2.

### Appendix A.1. Fringe Intensity Model and Modulation Depth Definition

For a phase-shifting fringe projection system, the captured intensity at each pixel can be written as

(A1) $$I=I_{r}+I_{m}\cos\phi$$

where $I_{r}$, $I_{m}$, and ϕ are defined in Equation (1) in Section 2.2 as the DC intensity, modulation amplitude, and fringe phase, respectively.

The modulation depth is defined as

(A2) $$k=\frac{I_{\max}-I_{\min}}{I_{\max}+I_{\min}}$$

For the sinusoidal model in Equation (A1),

(A3) $$I_{\max}=I_{r}+I_{m},\;I_{\min}=I_{r}-I_{m}$$

Substituting into Equation (A2) gives

(A4) $$k=\frac{I_{m}}{I_{r}}$$

Equation (A4) shows that the modulation depth is determined by the ratio between the AC amplitude and the DC intensity, which is a standard definition used in fringe projection profilometry and structured illumination imaging [10,12,25].

### Appendix A.2. Defect-Induced Perturbation of DC and AC Components

Local surface defects such as scratches, pits, scuffs, contamination, or roughness variations alter the local reflectance and scattering behavior. Consequently, both the DC component and the AC modulation amplitude may change:

(A5) $$I_{r}\to I_{r}+\Delta I_{r}$$

(A6) $$I_{m}\to I_{m}+\Delta I_{m}$$

where:

- $\Delta I_{r}$ represents the change in average reflectance;
- $\Delta I_{m}$ represents the change in modulation amplitude.

The perturbed modulation depth becomes

(A7) $$\tilde{k}=\frac{I_{m}+\Delta I_{m}}{I_{r}+\Delta I_{r}}.$$

### Appendix A.3. First-Order Perturbation Expansion

Assuming weak defect perturbations:

(A8) $$\left|\Delta I_{m}\right|\ll I_{m},\;\left|\Delta I_{r}\right|\ll I_{r}$$

Equation (A7) can be expanded using a first-order Taylor approximation:

(A9) $$\tilde{k}=\frac{I_{m}+\Delta I_{m}}{I_{r}+\Delta I_{r}}=k+\frac{\Delta I_{m}}{I_{r}}-k\frac{\Delta I_{r}}{I_{r}}+O\left({\left(\frac{\Delta I_{r}}{I_{r}}\right)}^{2},\;\frac{\Delta I_{m}}{I_{m}}\frac{\Delta I_{r}}{I_{r}}\right)$$

Using $k=I_{m}/I_{r}$, the perturbation can be written as

(A10) $$\tilde{k}=k+\Delta k+O\left(\epsilon^{2}\right).$$

where

(A11) $$\Delta k=\frac{\Delta I_{m}}{I_{r}}-k\frac{\Delta I_{r}}{I_{r}}$$

and

$$\epsilon =\max\left\{\left|\frac{\Delta I_{m}}{I_{m}}\right|,\left|\frac{\Delta I_{r}}{I_{r}}\right|\right\}$$

represents the perturbation magnitude.

Equation (A10) provides the theoretical justification of Equation (6) in the main text.

### Appendix A.4. Remainder Term and Validity of the Approximation

The neglected higher-order terms in Equation (A9) are therefore given by Equation (A12).

(A12) $$R=O\left({\left(\frac{\Delta I_{r}}{I_{r}}\right)}^{2},\;\frac{\Delta I_{m}}{I_{m}}\frac{\Delta I_{r}}{I_{r}}\right)$$

Notably, no standalone $O\left({\left(\Delta I_{m}/I_{m}\right)}^{2}\right)$ term appears in this ratio expansion, because the numerator perturbation enters linearly, whereas the second-order terms arise from the denominator expansion and the coupling between numerator and denominator perturbations. The neglected higher-order residual terms are summarized in Equation (A12), whose magnitude provides an estimate of the truncation error of the first-order expansion and thus characterizes the uncertainty introduced by the linear approximation. Under the weak-perturbation assumption commonly satisfied in practical inspection scenarios, these higher-order terms remain negligible compared with the first-order perturbation term.

Thus the additive perturbation model

(A13) $$\tilde{k}=k+\Delta k$$

is valid when the relative perturbations satisfy

(A14) $$\left|\frac{\Delta I_{m}}{I_{m}}\right|\ll 1,\;\left|\frac{\Delta I_{r}}{I_{r}}\right|\ll 1$$

These conditions correspond to weak defects or small reflectance/modulation variations, which are typical in early-stage industrial defect detection scenarios.

### Appendix A.5. Additive and Multiplicative Formulations

Equation (A13) describes the perturbation in an additive form. Introducing a dimensionless relative perturbation

(A15) $$\xi =\frac{\Delta k}{k}$$

the perturbed modulation depth can be written equivalently as

(A16) $$\tilde{k}=k\left(1+\xi \right)$$

Therefore:

- Equation (A13) expresses the absolute change in modulation depth;
- Equation (A16) expresses the relative (multiplicative) change.

Both formulations are mathematically equivalent and arise from the same perturbation expansion.

### Appendix A.6. Defect Severity and Imaging-Level Detectability

In principle, defect severity could be defined using physical parameters, such as:

- Scratch depth or width;
- Surface roughness variation;
- Scattering coefficient change.

However, these parameters depend strongly on material properties and defect types, making it difficult to define a unified physical severity scale.

Therefore, this work adopts an imaging-level defect severity (detectability) concept. The imaging-level defect severity adopted in this work is quantified using the background-noise-normalized saliency metric ${\tilde{V}}_{\mathrm{snr}}$, defined in Equation (13) in Section 3.3.2, which measures the detectability of defect responses relative to background fluctuations. This metric represents the signal-to-noise ratio of the defect response and is widely used in defect detection and visual saliency analysis [32,33,34,35,36].

Consequently,

(A17) $$S\equiv {\tilde{V}}_{\mathrm{snr}}$$

can be interpreted as an operational proxy for imaging-level defect severity.

Within the weak-perturbation regime of Equation (A14), the perturbation magnitude Δk and the detectability metric *S* are approximately monotonic. Thus the additive perturbation model in Equation (A13) provides a consistent link between physical reflectance perturbations and the statistical visibility metrics used in the experiments.

### Appendix A.7. Interpretation via Modulation Transfer Function (MTF)

The perturbation model derived above can also be interpreted from the perspective of the modulation transfer function (MTF) of the imaging system. As discussed in Section 3.6, the transmitted fringe modulation amplitude can be expressed as Equation (18): $M\left(f\right)=M_{0}\cdot \mathrm{MTF}\left(f\right)$ where $M_{0}$ denotes the intrinsic modulation amplitude of the projected fringe pattern and MTF(f) represents the combined optical transfer characteristics of the projector–camera system.

Consequently, the observed modulation amplitude can be written as

(A18) $$I_{m}=\mathrm{MTF}\left(f\right)\;I_{\mathrm{proj}}$$

where:

- $\mathrm{MTF}\left(f\right)$ is the modulation transfer function at spatial frequency *f*;
- $I_{\mathrm{proj}}$ is the projected fringe amplitude.

Local surface defects modify the local optical transfer characteristics through scattering, roughness variation, or reflectance changes. This effect can be modeled as a local perturbation of the modulation transfer function:

(A19) $$\mathrm{MTF}\left(f\right)\to \mathrm{MTF}\left(f\right)+\Delta \mathrm{MTF}\left(f\right)$$

Therefore the modulation amplitude becomes

(A20) $$I_{m}+\Delta I_{m}=\left(\mathrm{MTF}\left(f\right)+\Delta \mathrm{MTF}\left(f\right)\right)I_{\mathrm{proj}}$$

which yields

(A21) $$\frac{\Delta I_{m}}{I_{m}}=\frac{\Delta \mathrm{MTF}\left(f\right)}{\mathrm{MTF}\left(f\right)}$$

Substituting Equation (A21) into Equation (A11) gives

(A22) $$\Delta k=k\left(\frac{\Delta \mathrm{MTF}\left(f\right)}{\mathrm{MTF}\left(f\right)}-\frac{\Delta I_{r}}{I_{r}}\right)$$

Equation (A22) shows that the modulation perturbation arises from two physical mechanisms:

- Contrast attenuation caused by local MTF variations;
- Changes in average reflectance or illumination response.

This interpretation explains why the perturbation model in Equation (A13) provides a consistent description of defect-induced visibility changes in modulation images.
